# Comparison of PICU Cost and Severity-Adjusted Cost Between Patients With SIRS-Defined Sepsis and Those With Age-Adapted SOFA-Defined Sepsis

**DOI:** 10.3389/fped.2021.628918

**Published:** 2021-02-25

**Authors:** Xuepeng Zhang, Kaibo Sun, Guoyan Lu, Liwei Feng, Siyuan Chen, Yi Ji

**Affiliations:** ^1^Department of Pediatric Surgery, West China Hospital of Sichuan University, Chengdu, China; ^2^Department of Critical Care Medicine, West China Hospital of Sichuan University, Chengdu, China; ^3^West China School of Medicine, Sichuan University, Chengdu, China; ^4^Pediatric Intensive Care Unit, West China Second University Hospital, Sichuan University, Chengdu, China

**Keywords:** pediatric, sepsis, illness severity, critical care, cost

## Abstract

**Background:** The *2005 International Pediatric Sepsis Consensus* definition is considered to lack specificity and may lead to the admission of low-risk patients to the pediatric intensive care unit (PICU). The aim of this study was to compare the PICU cost and the severity-adjusted cost between patients with sepsis defined by the *2005 International Pediatric Sepsis Consensus* and those diagnosed using the age-adapted Sepsis-3 criteria.

**Methods:** Septic children identified by the *2005 Consensus* were screened for enrollment. The enrolled children were stratified into two subgroups using the age-adapted Sepsis 3.0 definition. A comparison was made between the subgroups of sepsis 3.0-defined children and non-sepsis 3.0-defined septic children. The Severity Adjusted ICU Cost (SAIC) was used to evaluate the case-mixed severity-adjusted costs of the study population. Coefficients in linear regression analyses in subgroups were calculated for presenting variation of PICU costs for every unit change of PRISM score.

**Results:** A total of 397 children were enrolled. The PICU length of stay was longer in the sepsis 3.0 group than in the non-sepsis 3.0 group [median (IQR), 9.0 (5.0, 15.0) vs. 6.0 (3.0, 9.0); *P* < 0.001]. Pediatric risk of mortality (PRISM) scores and mortality were significantly higher in sepsis 3.0-defined septic patients. The total costs and daily costs in the PICU were both significantly lower in the non-sepsis 3.0 group (*P* < 0.001). The severity-adjusted ICU cost in the non-sepsis 3.0 group was lower than that in the sepsis 3.0 group [median (IQR), 7,125 (3,588, 11,134) vs. 9,364 (5,680, 15,876); *P* = 0.001]. There was no significant difference among the regression coefficients.

**Conclusions:** The *2005 International Pediatric Sepsis Consensus* definition does not lead to more PICU costs after considering illness severity.

**Clinical Trial Registration:** www.ClinicalTrials.gov, identifier: NCT03598127.

## Introduction

The *2005 International Pediatric Sepsis Consensus* defines pediatric sepsis as an infection with ≥2 criteria for systemic inflammatory response syndrome (SIRS) (1). The definition of pediatric sepsis has not been updated for nearly 15 years, although evidence-based guidelines for the management of septic children have been published with great importance recently (2). The SIRS criteria for diagnosing sepsis are widely considered to lack sensitivity and specificity in adults (3, 4). In pediatric patients, the SIRS criteria also need to be validated. It was reported that the SIRS criteria were met or exceeded in more than 80% of infected children admitted to the PICU and in more than 90% of children with fever in the emergency department (5, 6). Then, it is inevitable that some septic children identified by the *2005 Consensus* with low mortality will be admitted to the PICU. Considering the high medical fees of the PICU, the issue of whether the medical costs of patients with low mortality match the illness severity is worthy of study. However, data on this issue are currently sparse.

In 2016, the Sepsis 3.0 consensus provided a better, high-mortality-recognizing definition that emphasized that sepsis is a life-threatening organ dysfunction resulting from a dysregulated host response to infection (7). According to the Sepsis 3.0 definition, an infected patient is diagnosed with sepsis when he/she has a Sequential Organ Failure Assessment (SOFA) score of two points or more. However, the criteria were developed for adult patients and were not suitable for children. Researchers previously adapted the Sepsis 3.0 definition for pediatric sepsis using age-adapted variables, and this approach showed better performance in recognizing pediatric patients with a high risk of mortality (5, 8).

In this study, we analyzed whether septic children identified by the *2005 International Pediatric Sepsis Consensus* definition have lower illness severity, although the PICU costs for these children do not concomitantly decrease. We compared the PICU cost and the severity-adjusted cost between SOFA- and non-SOFA-criteria septic children in the *2005 consensus*-defined septic population. In the *2005 Consensus*, severe sepsis was defined as sepsis + organ dysfunction, which is similar to the definition in Sepsis 3.0. Hence, we also evaluated the characteristics of children with severe sepsis.

## Methods

We performed an analysis among patients consecutively enrolled in a prospective cohort study registered on Clinicaltrials.gov (NCT03598127). The study was designed to investigate vitamin A levels in septic children and was conducted in the PICU at West China Hospital of Sichuan University, which is one of the largest academic hospitals located in western China. The study was approved by the ethics committee of the hospital (No. 2018-272). In this cohort, the patients received no additional intervention. All treatments were determined by physicians who were blind to the study. Parents of the enrolled children were informed about the study upon admission and provided written informed consent. All procedures were conducted in compliance with the Declaration of Helsinki.

Children who were diagnosed with sepsis upon PICU admission according to the *2005 International Pediatric Sepsis Consensus* from February 2018 to August 2019 were prospectively enrolled in this study. The enrolled children included patients in the emergency department and those who developed sepsis in general wards. The exclusion criteria were as follows: (1) children whose primary diagnosis for admission to the PICU was not sepsis; (2) patients who underwent surgery during their stay in the PICU since perioperative medical charges are associated with a large quantity of fees; (3) patients with underlying organ deficiency; and (4) patients older than 18 years of age.

In all eligible patients, we identified a subgroup of sepsis 3.0-defined septic children by using the age-adapted Sepsis 3.0 definition described in a previous study (5). Demographic data of all included patients were recorded upon admission. Pediatric Risk of Mortality I (PRISM I) scores were calculated by using the worst values in the first 24 h upon admission and were used to assess illness severity (9). The data for calculating the SOFA scores were collected in the first 24 h. Data on medical costs in the PICU were collected from the charge enquiry system in our hospital. All detailed charge information can be located in the system, and the system is open to patients. We defined the Severity Adjusted ICU Cost (SAIC) as the PICU cost divided by the PRISM severity score (9). The fundamental cost was defined as costs not related to illness severity, including blood gas analysis on admission, patient care once an hour by nurses, physical services, small consumables (such as bandages, gauzes, etc.), bed costs, central supply costs, costs of the monitor device for blood pressure/transcutaneous oxygen saturation/heart rate/respiratory rate, and professional services fees (physicians). These fees are charged once a patient has been admitted to the PICU regardless of his/her illness severity. Then, we calculated the SAIC using the following method:

$$\begin{array}{l} SAIC=\frac{C_{t}-F_{t}}{P}+F_{t}. \end{array}$$

Here, “*C*_*t*_” means total cost in the PICU, “*P*” represents PRISM scores calculated by using the worst values in the first 24 h, and “*F*_*t*_” represents the fundamental cost in the PICU. To avoid infinity, the “0” score was transformed to the “1” score when performing the adjustment. The SAIC was used to evaluate the case-mixed severity-adjusted costs of the study population. Student's *t*-tests or non-parametric tests (Mann-Whitney *U*-test) were used to compare the continuous variables, where appropriate, between the two groups. The chi-squared test or Fisher's exact test was used to analyze categorical data. Since illness severity scores are more useful in evaluating mortality in populations rather than individual patients, linear regression analyses were performed to evaluate the association between overall PRISM scores and PICU costs. In linear regression analysis, PRISM scores were independent variables and PICU costs were dependent variables. The coefficient β means that for every unit change of PRISM score, PICU cost will change by β units. Statistical analyses were performed using SPSS 22.0 for Windows (SPSS Inc., Chicago, IL, USA).

## Results

**Figure 1:**
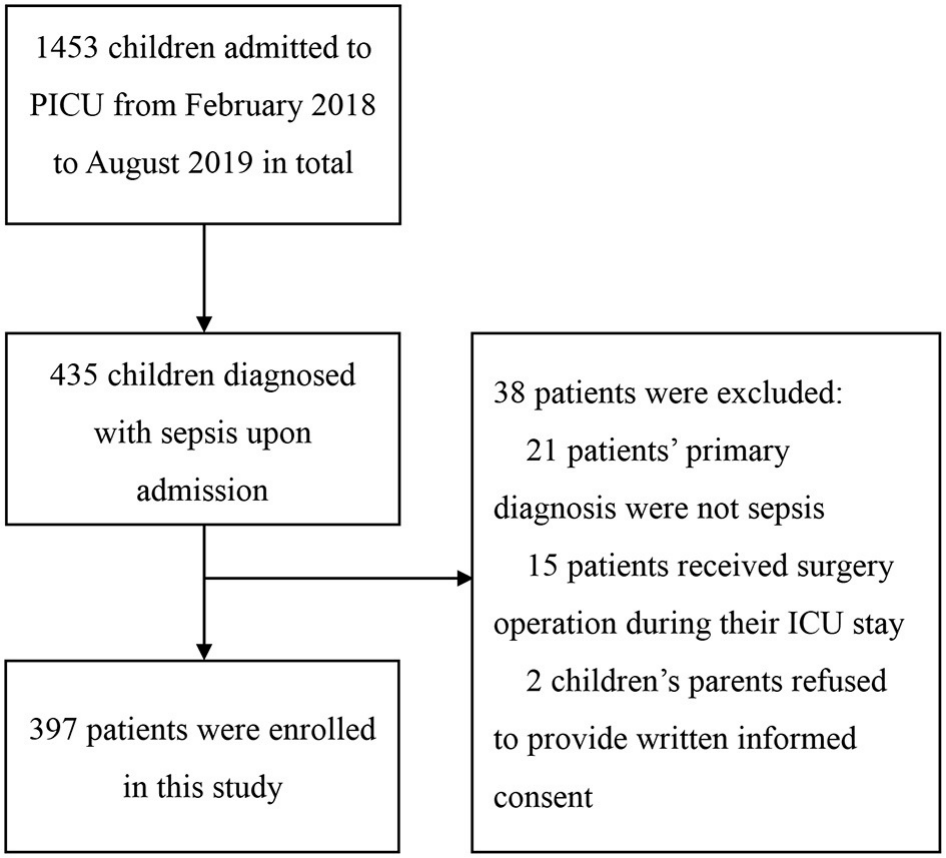
Flow of patient enrollment.

**Figure 2:**
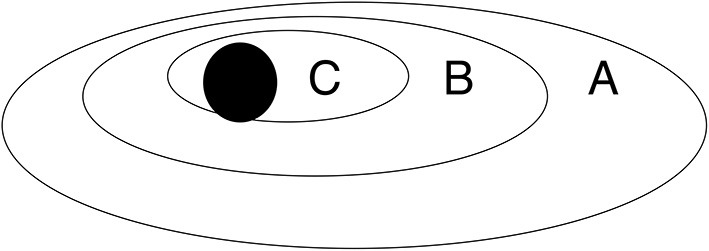
Area A represents the septic population identified by the *2005 International Pediatric Sepsis Consensus;* B represents children with sepsis who met the age-adapted Sepsis 3.0 criteria; C indicates patients with severe sepsis according to the *2005 International Pediatric Sepsis Consensus*; the black area represents death cases.

In total, 397 patients were prospectively enrolled in this study (Figure 1). The characteristics of the 397 included children are shown in Table 1. The median age was 9 months {median [interquartile range (IQR)], 9.00 [3.0, 42.50]}, and 218 of the children (54.91%) were boys. The overall mortality was 7.05%. The most frequent source of infection was respiratory infection. The median PRISM score was 8 [median (IQR), 8.0 (6.0, 13.0)]. The median PICU length of stay was 8.0 days [median (IQR), 8.0 (5.0, 14.0)]. There were 314 cases (79.09%) that met the age-adapted Sepsis 3.0 definition of sepsis and 83 cases (20.91%) that did not meet the definition. According to the *2005 Consensus*, 87 (21.91%) children met the criteria for severe sepsis. The 87 children were all included in the SOFA group. Figure 2 presents the relation of each subgroup. Differences in characteristics between patients with and without positive blood cultures were not significant (Supplementary Table 1).

**Table 1:** Baseline characteristics of the 397 children.

**Characteristics**	**Value**
Age, months	9.00 (3.0, 42.50)
Male, *n* (%)	218 (54.91%)
Source of infection, *n* (%)	
Respiratory system	234 (59.09%)
Gastrointestinal system	86 (21.72%)
Central nervous system	17 (4.29%)
Blood stream	22 (5.56%)
Soft tissue	10 (2.53)
Other	27 (6.82%)
Length of PICU stay, days	8.0 (5.0, 14.0)
Length of hospital stay, days	14.0 (9.0, 23.0)
PRISM	8.0 (6.0, 13.0)
SOFA	5.0 (2.0, 7.0)
Total PICU costs, yuan	28,038 (16,507, 54,055)
Total hospital costs, yuan	48,453 (28,974, 82,000)
Daily PICU costs, yuan	3,595 (2,929, 4,645)
Hospital mortality, *n* (%)	28 (7.05%)
Severe sepsis, *n* (%)	87 (21.91%)
Sepsis 3.0-defined sepsis	314 (79.09%)

*Data are presented as the median (interquartile range) or number (percentage)*.

*PICU, Pediatric Intensive Care Unit; PRISM, Pediatric Risk of Mortality score; SOFA, Sequential Organ Failure Assessment score*.

**Figure 3:**
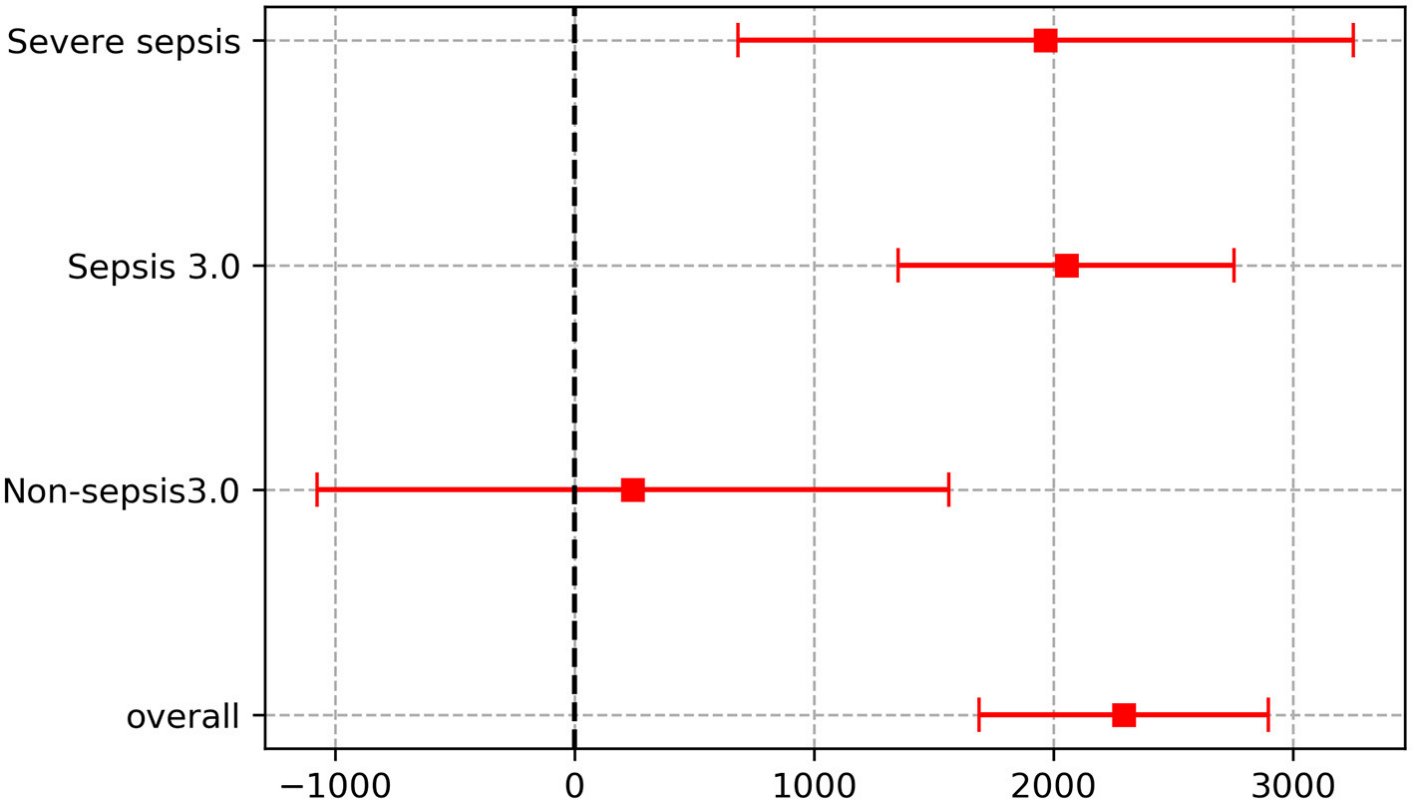
Distribution of each coefficients.

The differences in the distribution of age and sex between the sepsis 3.0 group and the non-sepsis 3.0 group were not significant (Table 2). Hospital length of stay and PICU length of stay were significantly longer in the sepsis 3.0 group [median (IQR), 15.50 (10.0, 24.0) vs. 12.0 (8.0, 16.0); *P* = 0.003 and 9.0 (5.0, 15.0) vs. 6.0 (3.0, 9.0); *P* < 0.001, respectively]. PRISM scores in sepsis 3.0-defined septic patients were significantly higher than those in the rest of the children [median (IQR), 9.0 (5.0, 15.0) vs. 6.0 (3.0, 9.0); *P* < 0.001]. The sepsis 3.0 group had higher hospital mortality than the non-sepsis 3.0 group, and the difference was statistically significant (8.60% vs. 1.20%, *P* = 0.019). The total PICU cost, hospital cost, and daily PICU cost in the sepsis 3.0 group were significantly higher than the costs in the non-sepsis 3.0 group (Table 2). After adjusting for PRISM scores, the PICU cost in the non-sepsis 3.0 group was lower than that in the sepsis 3.0 group [median (IQR), 7,125 (3,588, 11,134) vs. 9,364 (5,680, 15,876); *P* = 0.001]. Table 3 shows the coefficients in each linear regression analysis. The coefficient of PRISM scores in regression analysis in non-sepsis 3.0 group was not higher than the coefficient in sepsis 3.0 group (Figure 3).

**Table 2:** Comparison of patients with and without sepsis-3 definition sepsis.

**Characteristics**	**Sepsis-3 *N* = 314**	**Non-sepsis-3*N* = 83**	***P***
Age, months	8.50 (2.0, 44.0)	14.0 (4.0, 36.0)	0.145
Male, *n* (%)	169 (53.82%)	49 (59.04%)	0.468
PRISM	9.0 (6.0, 15.0)	5.0 (4.0, 8.0)	0.000^a^
SOFA	6.0 (4.0, 8.0)	1.0 (1.0, 1.0)	0.000^a^
Total PICU cost, yuan	32,118 (19,249, 65,381)	18,171 (9,605, 27,913)	0.000^a^
Total hospital cost, yuan	52,571 (32,061, 94,190)	31,843 (22,186, 52,129)	0.000^a^
Daily PICU cost, yuan	3,727 (3,042, 4,966)	3,210 (,2550, 3,795)	0.001
SAIC	9,364 (5,680, 15,876)	7,125 (3588, 11,134)	0.001
PICU stay, days	9.0 (5.0, 15.0)	6.0 (3.0, 9.0)	0.000^a^
Hospital stay, days	15.50 (10.0, 24.0)	12.0 (8.0, 16.0)	0.003
Mortality, *n* (%)	27 (8.60%)	1 (1.20%)	0.019

*Data are presented as the median (interquartile range) or number (percentage)*.

*PICU, Pediatric Intensive Care Unit; PRISM, Pediatric Risk of Mortality score; SOFA, Sequential Organ Failure Assessment score; SAIC, Severity Adjusted ICU Cost*.

a *P < 0.001*.

**Table 3:** Coefficients in each linear regression analysis.

**Group**	**Coefficients**	**95% CI**
Overall	2291.958	1689.409–2894.506
Non-sepsis3.0	244.357	−1074.552–1563.265
Sepsis 3.0	2052.157	1352.268–2752.047
Non-severe sepsis	1249.663	183.799–2315.527
Severe sepsis	1966.055	681.638–3250.472

*CI, Confidence Interval*.

Table 4 lists the characteristics of the 87 children with severe sepsis identified by the *2005 Consensus*. Twenty-four (27.59%) patients in the severe sepsis subgroup died. The PRISM and SOFA scores were significantly higher in severe sepsis patients [median (IQR), 14.0 (8.0, 23.0) vs. 8.0 (6.0, 12.0); *P* < 0.001]. Total PICU costs and daily costs were higher in severe sepsis patients (*P* = 0.013 and *P* < 0.001, respectively), but the differences in total hospital stay between the two subgroups were not significant. The distributions of length of PICU stay, length of hospital stay, and SAIC were similar between the two subgroups.

**Table 4:** Comparison between patients with and without severe sepsis in sepsis-3 definition patients.

**Characteristics**	**Severe sepsis *N* = 87**	**Non-severe sepsis*N* = 227**	***P***
Age, months	12.0 (2.0, 53.0)	7.0 (3.0, 42.0)	0.207
Male, *n* (%)	48 (55.17%)	122 (53.70%)	0.820
PRISM	14.0 (8.0, 23.0)	8.0 (6.0, 12.0)	0.000
SOFA	8.0 (5.0, 10.0)	5.0 (4.0, 7.0)	0.000
Total PICU cost, yuan	44,869 (19,979, 109,360)	29,879 (19,142, 56,333)	0.013
Total hospital cost, yuan	63,732 (30,608, 130,067)	51,587 (32,209, 85,860)	0.056
Daily PICU cost, yuan	4,259 (3,603, 7,162)	3,612 (2,978, 4,723)	0.000
SAIC	9,198 (5,680, 19,724)	9,464 (5,680, 15,322)	0.522
PICU stay, days	9.0 (5.0, 22.0)	9.0 (5.0, 14.0)	0.496
Hospital stay, days	17.0 (8.0, 29.0)	15.0 (10.0, 23.0)	0.901
Mortality, *n* (%)	24 (27.59%)	3 (1.30%)	0.000

*Data are presented as the median (interquartile range) or number (percentage)*.

*PICU, Pediatric Intensive Care Unit; PRISM, Pediatric Risk of Mortality score; SOFA, Sequential Organ Failure Assessment score; SAIC, Severity Adjusted PICU Cost*.

## Discussion

Our data revealed that in the cohort, the non-sepsis 3.0 group had lower hospital mortality and shorter stays in the PICU and hospital than the sepsis 3.0 group. These findings suggest that the *2005 Pediatric Sepsis Consensus* definition may lack specificity in recognizing patients with a high risk of mortality compared to the Sepsis 3.0 criteria. Previous epidemiologic studies on septic children who were diagnosed by the *2005 Pediatric Sepsis Consensus* reported that mortality in these children was similar to or even lower than mortality in the general PICU population (10–13). The most plausible reason is that the SIRS criteria lack specificity since the *2005 Pediatric Sepsis Consensus* is based on the SIRS criteria. Many pediatric patients presented with fever, tachycardia, and/or tachypnea and met the SIRS diagnostic criteria. Some diseases (e.g., bronchiolitis) had very low mortality or even near-zero mortality. However, the patients may have been diagnosed with pediatric sepsis and therefore may have been admitted to the PICU. Another study in a large pediatric population that evaluated the SIRS criteria also revealed that SIRS criteria could increase the risk of PICU admission (RR = 1.45), but the criteria were not associated with hospital mortality (6).

In the current study, 20% of septic children identified by the *2005 Pediatric Sepsis Consensus* had lower mortality and less illness severity. The total PICU cost and daily PICU cost were significantly lower in the non-SOFA group. Considering the impact of illness severity on cost, the SAIC in the non-SOFA group was still lower than that in the SOFA group. These findings suggest that some septic patients admitted to the PICU may not be at high risk of mortality, but the financial costs of the PICU for these patients are relatively lower. Furthermore, coefficients in linear regression analyses performed in the subgroups also showed no significant difference. Those findings do not support our hypothesis that the cost expenditures for children who were admitted to the PICU with *2005 consensus*-identified sepsis may not match the illness severity. This could be explained by the fact that few patients in the non-sepsis 3.0 group suffered organ dysfunction and received organ support therapy, such as ventilation and continuous renal replacement therapy. More importantly, those patients had a shorter length of PICU stay.

Sepsis involves a high risk of mortality. It threatens millions of people's lives each year globally, and tremendous resources are expended to improve the outcome of patients with sepsis (7). Our findings of the current study suggest that the SOFA-based sepsis definition may be better in identifying septic children with a high risk of mortality than the *2005 Consensus* definition. The definition of severe sepsis in the *2005 Consensus* is similar to Sepsis 3.0 and seems more specific in recognizing high-risk patients, but it may lack sensitivity. In this study, the severe sepsis subgroup had the highest severity scores and highest mortality. However, this group may exclude some children at high risk of mortality. Some septic children with organ injuries may not initially meet the severe sepsis criteria but have the potential to develop organ dysfunction, and these patients may be excluded by the severe sepsis criteria.

Another interesting finding is that the non-SOFA group was older than the SOFA group, although the difference was not statistically significant. Previous studies revealed a similar result: younger children are more vulnerable to infection than older children, leading to aggravation of the severity of disease (13). This finding may also indicate that the SIRS-based pediatric sepsis definition does not perform well in recognizing septic children with a high risk of mortality.

This study has several limitations. First, the major limitation of the study is that a comparison was made between two subsets of patients defined by the *2005 Pediatric Sepsis Consensus*. The characteristics of the children who met the SOFA-based sepsis criteria but not the SIRS-based criteria were unknown, which is the most important source of bias. Second, there were no non-SOFA septic children from general wards to serve as a control group. The medical costs and mortality of the non-SOFA group in general wards were unclear. In addition, the new SAIC indicator that we first proposed may not be very accurate since the illness severity scores vary in different phases of sepsis. However, it is impossible to ensure that all patients are admitted in the same phase of disease. Furthermore, severity scores varied during the PICU stay, and we failed to calculate the daily PRISM scores in the PICU in this cohort and adjusted total PICU costs by PRISM scores on day 1. The following equation may be more accurate:

$$\begin{array}{l} SAIC=\overset{M}{\underset{j=1}{\sum }}\left(\frac{C_{j}-F_{j}}{P_{j}}+F_{j}\right). \end{array}$$

Here, “*j*” means day “*j*” in the PICU, and “*M*” represents the length of PICU stay. Finally, this was a single-center study, which may have led to selection bias.

## Conclusion

The *2005 International Pediatric Sepsis Consensus* definition may not lead to more PICU costs after considering illness severity, though the *2005 Consensus* may lack specificity in recognizing children with a high risk of mortality compared to Sepsis 3.0.

## Data Availability Statement

The raw data supporting the conclusions of this article will be made available by the authors, without undue reservation.

## Ethics Statement

The studies involving human participants were reviewed and approved by the Ethics Committee of West China Hospital. Written informed consent to participate in this study was provided by the participants' legal guardian/next of kin.

## Author Contributions

SC, YJ, and XZ designed the study. LF and GL collected data and managed its quality. XZ and KS performed the statistical analysis and drafted the manuscript. SC and YJ contributed substantially to its revision. All authors participated data interpretation, read the manuscript carefully, and approved the final version.

## Conflict of Interest

The authors declare that the research was conducted in the absence of any commercial or financial relationships that could be construed as a potential conflict of interest.

## Abbreviations

SIRS: Systemic Inflammatory Response Syndrome
SOFA: Sequential Organ Failure Assessment
PICU: Pediatric Intensive Care Unit
PRISM: Pediatric Risk of Mortality
SAIC: Severity Adjusted PICU Cost.
