# Supplementary material for: Comparison of PICU Cost and Severity-Adjusted Cost Between Patients With SIRS-Defined Sepsis and Those With Age-Adapted SOFA-Defined Sepsis
Source: Front Pediatr. 2021 Feb 25;9:628918. doi: 10.3389/fped.2021.628918 (PMC7947209; doi:10.3389/fped.2021.628918)
Supplement: Supplementary file 1 [file Table_1.docx]

**Supplementary table 1**. Characteristics of positive blood culture and negative blood culture

|  | Positive culture  N= 22 | Negative culture  N=375 | *P* |
| --- | --- | --- | --- |
| Age, months | 37.0 (4.75, 74.50) | 9.0 (3.0, 39.0) | 0.359 |
| Male, n (%) | 10 (45.50%) | 208 (55.50%) | 0.468 |
| PRISM | 10.0 (5.50, 16.50) | 8.0 (6.0, 13.0) | 0.550 |
| SOFA | 6.0 (3.0, 8.0) | 5.0 (2.0, 7.0) | 0.175 |
| Total PICU cost, yuan | 23474 (17520, 72277) | 28163 (16489, 52964) | 0.782 |
| Total hospital cost, yuan | 60460 (28342, 133115) | 48294 (29191, 81421) | 0.288 |
| Daily PICU cost, yuan | 3814 (2257, 5573) | 3586 (2931, 4602) | 0.945 |
| SAIC | 8449 (6036, 24783) | 8446 (5406, 14988) | 0.512 |
| PICU stay, days | 7.0 (4.0, 21.25) | 8.0 (5.0, 14.0) | 0.877 |
| Hospital stay, days | 18.50 (11.75, 27.0) | 14.0 (9.0, 22.0) | 0.062 |
| Mortality, n (%) | 3 (13.60%) | 25 (6.70%) | 0.196 |

Data are presented as the median (interquartile range) or number (percentage).

Abbreviations: PICU, Pediatric Intensive Care Unit; PRISM, Pediatric Risk of Mortality score; SOFA, Sequential Organ Failure Assessment score; SAIC, Severity Adjusted ICU Cost
